# Effect of the JAK Inhibitor Baricitinib on Cytokine Production and Bone Properties in a Mouse Model of Accelerated Aging

**DOI:** 10.3390/ijms27115047

**Published:** 2026-06-03

**Authors:** Katharina Gelles, Vincent Kurz, Maria Butylina, Katharina Wahl-Figlash, Martin Schepelmann, Anastasia Meshcheryakova, Peter Pietschmann

**Affiliations:** 1 Institute of Pathophysiology and Allergy Research, Center for Pathophysiology, Infectiology and Immunology, Medical University of Vienna, 1090 Vienna, Austria; katharina.gelles@meduniwien.ac.at (K.G.); vinc.yoshi@gmail.com (V.K.); maria.butylina@meduniwien.ac.at (M.B.); katharina.wahl@meduniwien.ac.at (K.W.-F.); martin.schepelmann@meduniwien.ac.at (M.S.); 2 Comprehensive Center for AI in Medicine (CAIM), Medical University of Vienna, 1090 Vienna, Austria

**Keywords:** osteoporosis, JAK inhibition, baricitinib, SAMP8, cytokines, µCT, histomorphometry

## Abstract

Age-related osteoporosis is characterized by progressive loss of bone mass and deterioration of bone microarchitecture, leading to enhanced skeletal fragility. Cytokines regulate bone remodeling through distinct signaling pathways. Baricitinib, a selective JAK1/2 inhibitor effective in inflammatory disorders such as rheumatoid arthritis, suppresses cytokine signaling, but its role in age-related osteoporosis remains insufficiently defined. In our study a total of 60 eight-month-old female SAMP8 mice were randomized to receive baricitinib (10 mg/kg) or vehicle twice daily by oral gavage for six weeks. Bone outcomes were evaluated by high-resolution micro-computed tomography (µCT) and static histomorphometry. Intracellular cytokine production by splenocytes was determined via flow cytometry. We found that baricitinib substantially reduced T-cell cytokine production, decreasing IL-6, IL-17, IFN-γ, and IL-21 in CD4^+^ T cells and IL-6 in CD8^+^ T cells, accompanied by lower IFN-γ/IL-17 and IL-21/IL-6 ratios, respectively. µCT analyses showed no significant intergroup differences in BV/TV, whereas histomorphometry demonstrated higher BV/TV in the baricitinib group. Overall, baricitinib was found to effectively suppressed proinflammatory cytokines in aged SAMP8 mice but did not consistently enhance bone parameters, indicating reduced skeletal responsiveness during aging.

## 1. Introduction

Osteoporosis is a progressive age-related disease, leading to increased porosity and weakness of bones [1]. It is characterized by a systemic impairment of bone mass, increased skeletal fragility, and microarchitectural deterioration of bone tissue [2,3]. With advancing age, an imbalance in bone metabolism results in a dysregulation of osteoblast and osteoclast activity, leading to an increased bone resorption but decreased bone formation [4,5,6]. A concomitant decline in sex hormones promotes osteoclast activity and accelerates bone loss, which contributes to the higher prevalence of osteoporosis in postmenopausal women compared to men [3,4]. Osteoporosis is responsible for more than nine million fractures every year, and the risk of further fractures is markedly elevated for those who have already suffered an osteoporotic fracture. However, in clinical practice, only a minority of patients currently receive appropriate treatment, which highlights the urgent need for novel therapeutic approaches [1,5,7,8,9].

In the 1970s, researchers were already aware of the close connection between the immune and skeletal system, which led to the introduction of the term osteoimmunology in the year 2000 [10,11]. Bone loss is a hallmark of autoimmune and inflammatory diseases and related to a dysregulation of cytokines, resulting in enhanced osteoclastogenesis, while osteoblast activity is impaired. Cytokines are structurally distinct soluble factors that play a key role in bone homeostasis; via their signaling pathways, they influence generation and activation of osteoclasts and consequently regulate bone resorption [12,13,14,15,16].

For several cytokines, the Janus kinase (JAK) and signaling transducer of activators of transcription (STAT) system serves as a pathway for the transduction of intracellular signals [15,17]. JAK and STAT knockout models suggest that the JAK/STAT pathway also plays a key role in skeletal development [18]. JAKs are intracellular, non-receptor tyrosine kinases; upon cytokine binding to their respective receptors, JAKs become enzymatically activated [17,19]. The success of therapeutic approaches not only targeting cytokines and their receptors, but also intracellular pathways affected by cytokines, highlights their central role in driving immune-mediated diseases [16,20]. Since the mid-2010s, highly specific JAK inhibitors (JAKis), small molecule inhibitors targeting the JAK family kinases, have been approved for the treatment of inflammatory and hematological diseases [15,17,19]. Currently, more than 14 JAKis have received approval by major regulatory agencies for use across multiple indications; four JAK inhibitors are available for the treatment of RA, which is characterized by a serious decline in bone mass, namely tofacitinib, baricitinib, upadacitinib and filgotinib [15,17,21,22]. Baricitinib is an orally administered, selective small molecule inhibitor of JAK1/JAK2. With its anti-inflammatory properties, it has shown clinical efficacy in the treatment of RA by inhibiting structural joint damage and mitigating bone erosions under inflammatory conditions [15,23,24,25]. Furthermore, baricitinib inhibits osteoclastogenesis in vitro by reducing the osteoblast-derived receptor for activation of nuclear factor kappa B (NF-κB) ligand (RANKL) expression; in vivo, oral treatment was shown to attenuate inflammation and bone resorption in rodent arthritis models. Overall, baricitinib suppresses osteoclast formation and may therefore help prevent bone resorption [14,26,27]. However, the effects of baricitinib on bone mineral density (BMD) and the overall effects of JAK inhibition on bone are not well defined [15,28]. Adam et al. [15] demonstrated in 2020 that JAK inhibition with baricitinib or tofacitinib increases trabecular bone volume fraction in healthy young C57BL/6 mice (11 weeks old) and ovariectomized mice (16 weeks old), and improves both cortical and trabecular bone parameters in an arthritis model. Based on in vitro data, the authors further indicate that JAK inhibitors promote bone formation via the Wnt (Wingless-related integration site) signaling pathway, supporting their function as bone-active compounds.

In this study we investigated the effects of baricitinib on age-related osteoporosis. We aimed to determine whether baricitinib leads to an improvement in the bone properties of the senescence-accelerated mice-prone 8 (SAMP8), a mouse model of accelerated aging, and evaluated the effects of baricitinib on bone metabolism The SAMP8 mouse serves as a model of age-related osteoporosis with co-existing sarcopenia [29]. These mice show characteristic signs of accelerated aging, including reduced muscle mass, a shortened lifespan, and progressive oxidative-stress-related aging [30]. Furthermore, SAMP8 mice represent a pre-sarcopenic stage at 8 months of age and develop sarcopenia by 10 months, providing a model in which osteoporotic fracture healing is impaired [29,30].

## 2. Results

### 2.1. No Effect of Baricitinib Treatment on Survival and Bodyweight

**Figure 1:**
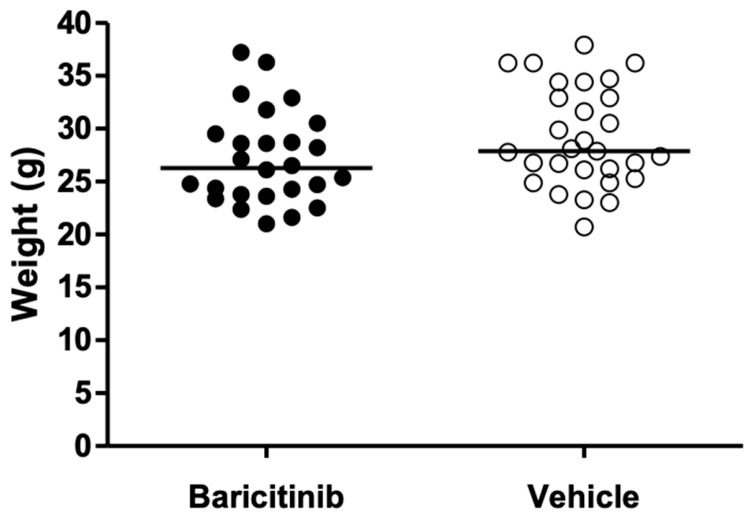
Body weight of baricitnib-treated and vehicle-treated SAMP8 mice at the day of sacrifice. Group comparison was performed using the Mann–Whitney U test. Each dot represents a single mouse. Horizontal lines indicate the median.

During the treatment period, the body weight of SAMP8 mice treated with baricitinib was comparable to vehicle-treated mice (Figure 1). No statistically significant difference was found.

### 2.2. Baricitinib Treatment Profoundly Alters Cytokines Associated with Inflammation and Bone Turnover

**Figure 2:**
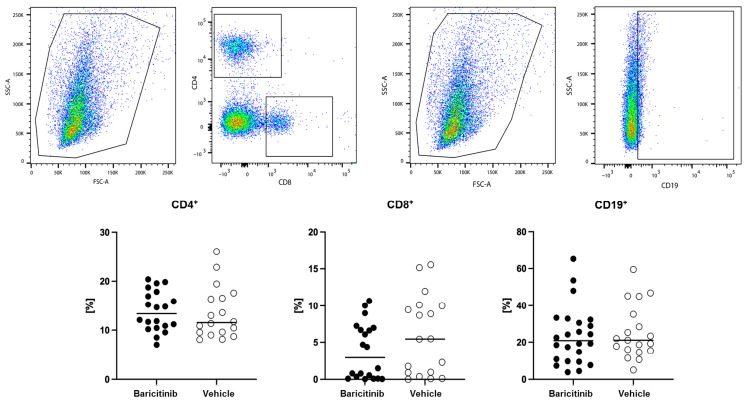
Gating strategy and frequencies of CD4^+^, CD8^+^, and CD19^+^ cells. In the first step, the FSC/SSC blot was used to differentiate between living cells and cell debris. Next, gating was performed on the basis of the CD8/CD4 blot to dissect CD8^+^ and CD4^+^ cells, and on the basis of the CD19/SSC blot to dissect CD19^+^ cells. Pseudocolor plots are used to visualize the density of cell population(s). Representative dot plots illustrating the gating strategy are shown, as well as the frequencies of CD4^+^, CD8^+^, CD19^+^ cells for the baricitinib-treated and the vehicle-treated group.

**Figure 3:**
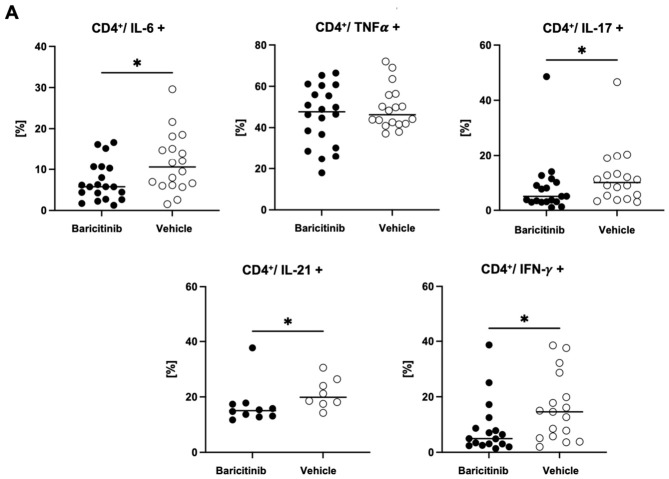

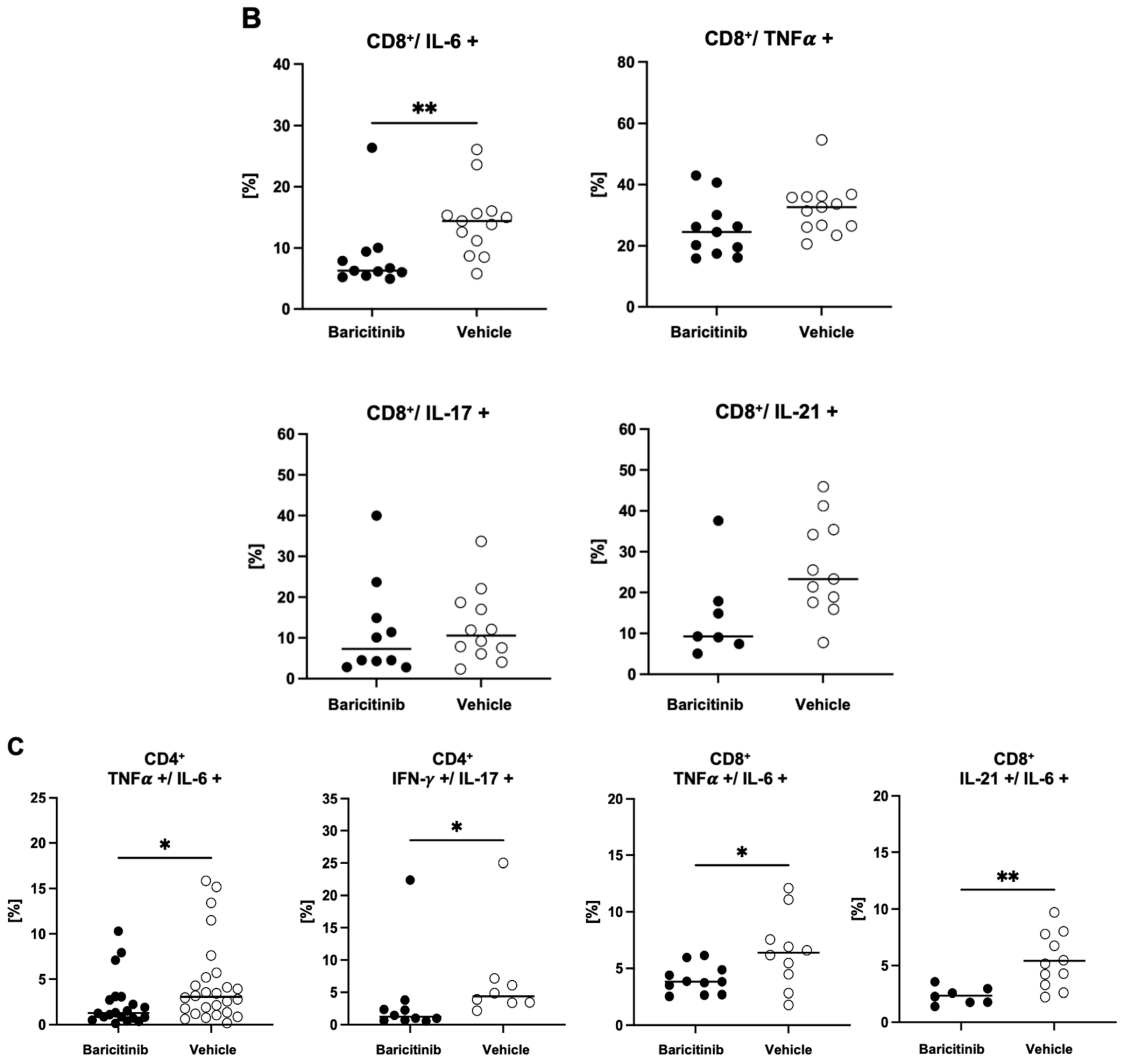
Evaluation of the intracellular cytokine levels by flow cytometry. Scatter dot plots illustrate the levels of (**A**) IL-6 (*p* = 0.034), TNFα, IL-17 (*p* = 0.033), IL-21 (*p* = 0.032) and IFN-γ (*p* = 0.021) in CD4^+^ T cells, and (**B**) IL-6 (*p* = 0.005), TNFα, IL-17 and IL-21 in CD8^+^ T cells. Data are not normally distributed; the Mann–Whitney U test was used except for TNFα in CD4^+^ and CD8^+^ T cells and IL-21 in CD8^+^ T cells; here, unpaired t-tests were used. (**C**) Scatter dot plots illustrate the cytokine levels in CD4^+^, including TNFα +/IL-6 + (*p* = 0.026), IFN-γ +/IL-17 + (*p* = 0.012) and CD8^+^ T cells, including TNFα +/IL-6 + (*p* = 0.029), IL-21 +/IL-6 + (*p* = 0.005). Data are not normally distributed; the Mann–Whitney U test was used except for IL-21 +/IL-6 + in CD8^+^ T cells; here, unpaired *t*-tests were used. * *p* < 0.05, ** *p* < 0.01. Horizontal lines indicate the median.

**Figure 4:**
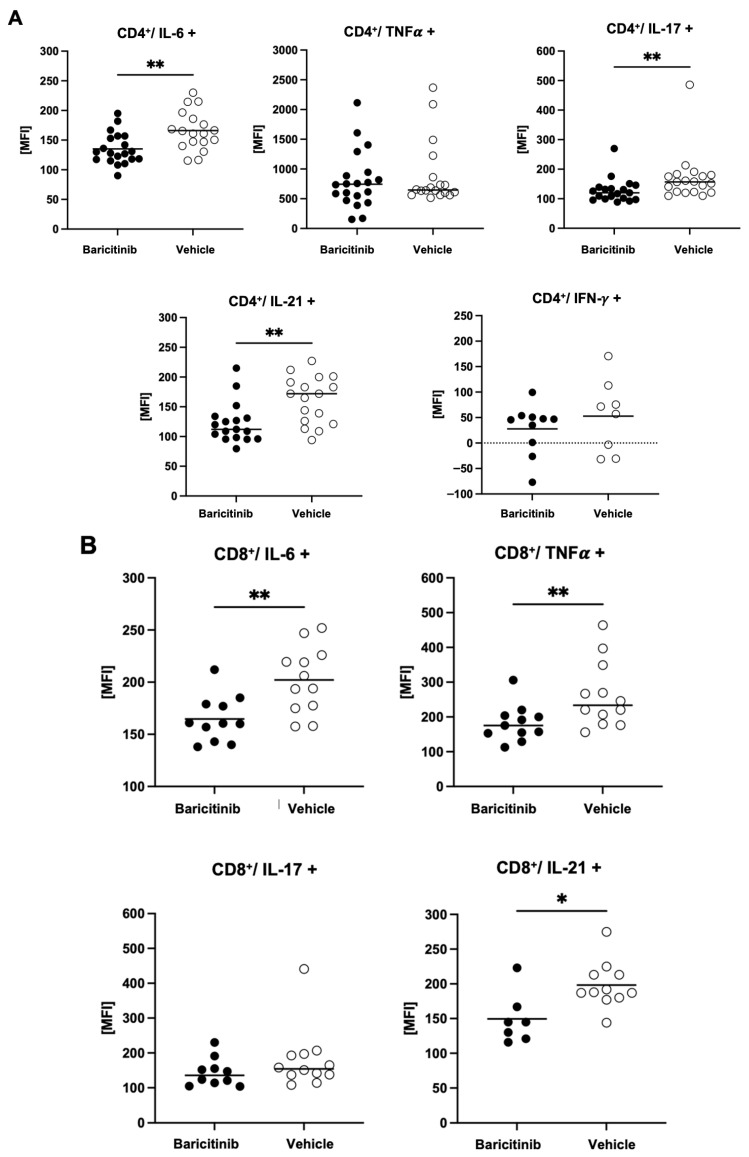
Evaluation of the MFI of intracellular cytokine levels. (**A**) Scatter dot plots illustrate mean fluorescence intensity (MFI) attributed to IL-6 (*p* = 0.003), TNFα, IL-17 (*p* = 0.003), IL-21 (*p* = 0.007), and IFN-γ in CD4^+^ T cells, and (**B**) IL-6 (*p* = 0.004), TNFα (*p* = 0.009), IL-17, and IL-21 (*p* = 0.010) in CD8^+^ T cells. Data are not normally distributed; the Mann–Whitney was used except for IL-6 and IFN-γ in CD4^+^ and IL-6 and IL-21 in CD8^+^ T cells; here, unpaired *t*-tests were used. * *p* < 0.05, ** *p* < 0.01. Horizontal lines indicate the median.

To determine whether baricitinib influenced immune-related cytokine production, we analyzed the levels of interleukin-6 (IL-6), TNFα, IL-17, IL-21 and interferon-γ (IFN-γ) in splenic immune cell populations of SAMP8 mice. We did not detect any changes in the frequencies of CD4^+^ and CD8^+^ T cells and CD19^+^ B cells upon baricitinib treatment (Figure 2). We found a significant reduction in IL-6, IL-17, IL-21 and IFN-γ levels in CD4^+^ T cells (Figure 3A) and of IL-6 levels in CD8^+^ T cells (Figure 3B) in the baricitinib-treated group in comparison to the vehicle group. Furthermore, group comparison revealed a statistically significant reduction in TNFα/IL-6 and IFN-γ/IL-17 levels in CD4^+^ T cells and of TNFα/IL-6 and IL-21/IL-6 levels in CD8^+^ T cells (Figure 3C). The analysis of cytokine combinations focused on cell populations that express both cytokines simultaneously. The analysis of mean fluorescence intensity (MFI) values showed reduced cellular expression levels of IL-6, IL-17 and IL-21 in CD4^+^ T cells (Figure 4A), and of IL-6, TNFα and IL-21 in CD8^+^ T cells (Figure 4B). We observed a decrease in both the percentage of cells producing the indicated proinflammatory cytokines as well as in the MFI values, which represent the level of cytokine expression per cell. Given that, we can conclude that baricitinib reduces the number of cells producing the proinflammatory cytokines, as well as the level of production per cell. Frequencies of CD4^+^ T cells and CD8^+^ T cells and CD19^+^ B cells were furthermore evaluated in bone marrow (Supplementary Figure S1); no statistical significance between the baricitinib-treated group and vehicle-treated group was found.

Overall, the data showed a profound effect of baricitinib on the expression of cytokines related to inflammation and bone turnover.

### 2.3. No Effect of Baricitinib Treatment on Serum Bone Turnover Markers

**Figure 5:**
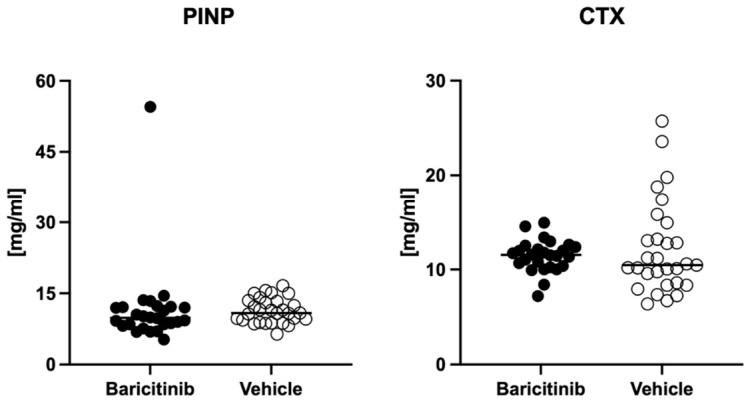
Bone turnover markers. Scatter dot plots show serum levels of PINP and CTX in baricitinib-treated (*n* = 26) and vehicle-treated (*n* = 29) SAMP8 mice. All data are not normally distributed; the Mann–Whitney U test was used. Horizontal lines indicate the median.

Serum markers of bone turnover p-terminal propeptide of type I collagen (PINP) and c-terminal telopeptide of type I collagen (CTX) levels did not differ significantly between baricitinib-treated and vehicle-treated SAMP8 mice after 6 weeks of treatment. In the vehicle group, serum CTX showed a wider variation and several values above the highest value of the baricitinib group (Figure 5).

### 2.4. Femoral and Vertebral µCT Parameters Were Not Affected by Baricitinib Treatment

**Figure 6:**
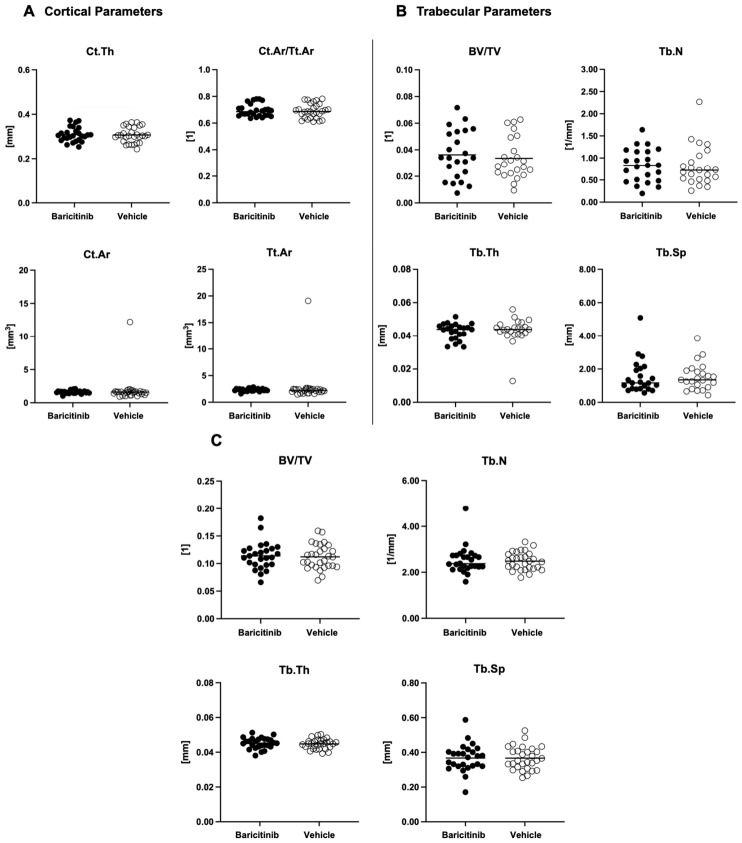
µCT analysis of the femur and the fourth vertebral body. Parameters derived from the femur are shown for baricitinib-treated (*n* = 26) and vehicle-treated (*n* = 29) SAMP8 mice. (**A**) Cortical parameters: average cortical thickness (Ct.Th), cortical bone area fraction (Ct.Ar/Tt.Ar), cortical bone area (Ct.Ar), total cross-sectional area (Tt.Ar). (**B**) Trabecular parameters: bone volume/total volume (BV/TV), trabecular number (Tb.N), trabecular thickness (Tb.Th), trabecular separation (Tb.Sp). (**C**) Parameters of the lumbar spine are presented as follows: bone volume/total volume (BV/TV), trabecular number (Tb.N), trabecular thickness (Tb.Th), trabecular separation (Tb.Sp). Data are not normally distributed; Mann–Whitney was used except for Ct.Ar and BV/TV in the femur and BV/TV, Tb.Th and Tb.Sp in the vertebral body; here, unpaired *t*-tests were used. Horizontal lines indicate the median.

Microstructural measurements of the femur revealed no significant differences between the baricitinib and vehicle group across the assessed parameters (Figure 6A,B). This includes the cortical parameters Ct.Th, Ct.Ar/Tt.Ar, Ct.Ar and Tt.Ar. Trabecular parameters of the femur were likewise comparable: BV/TV, Tb.N, Tb.Th and Tb.Sp did not differ between the groups.

For the fourth vertebral body, no differences were found for any of the trabecular parameters such as BV/TV, Tb.N, Tb.Th and Tb.Sp when comparing baricitinib with the vehicle group (Figure 6C). Overall dispersion was similar between groups.

### 2.5. Minor Effect of Baricitinib Treatment on Vertebral Bone Microarchitecture

**Figure 7:**
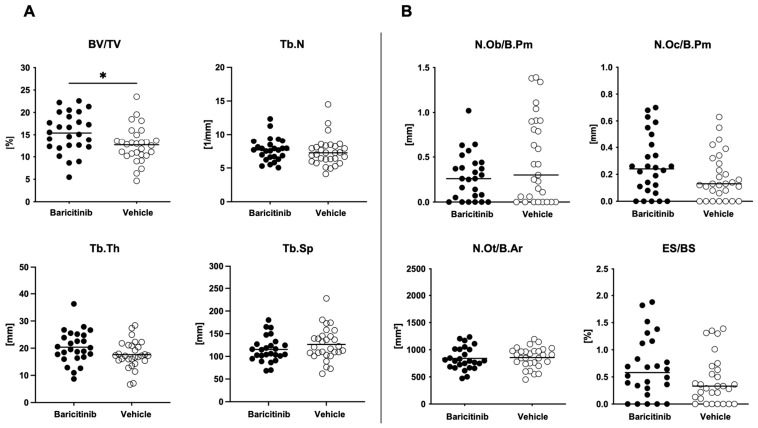
Bone histomorphometry of the fifth vertebral body. Trabecular and static bone histomorphometric parameters are shown: (**A**) bone volume/total volume (BV/TV) *p* = 0.024, trabecular number (Tb.N), trabecular thickness (Tb.Th), trabecular separation (Tb.Sp); (**B**) number of osteoblasts/bone perimeter (N.Ob/B.Pm), number of osteoclasts/bone perimeter (N.Oc/B.Pm), number of osteocytes/bone area (N.Ot/B.Ar), and eroded surface/bone surface (ES/BS). Group comparison of baricitinib-treated (*n* = 26) and vehicle-treated (*n* = 29) SAMP8 mice. Each dot represents one animal. * *p* < 0.05. Data are not normally distributed; Mann–Whitney U was used except for Tb.Th, Tb.Sp and N.Ot/B.Ar; here, unpaired *t*-tests were used. Mean values are used for normally distributed and median values for non-normally distributed data. Horizontal lines indicate the median.

However, in contrast to µCT measurements, we observed a small effect of baricitinib treatment on bone microarchitecture in the fifth vertebral body as analyzed by static histomorphometry, as BV/TV was significantly increased in SAMP8 mice treated with baricitinib compared to the vehicle group (Figure 7A,B). All other measured parameters did not differ between the two groups. Dynamic histomorphometry was performed via calcein double-labeling to assess MAR, MS/BS and BFR/BS and to confirm the aforementioned results of the µCT evaluations. For this type of analysis, five mice per group were used. Across all measured parameters, results were variable and did not show significant differences between the baricitinib and vehicle group. Non-normally distributed data are represented as median with interquartile range [Q1–Q3]; normally distributed data as mean with standard deviation: MAR (baricitinib: 0.694 ± 0.886, vehicle: 0.2797 ± 0.310), MS/BS (baricitinib: 1.449 [0.4536–7.008], vehicle: 0.4189 [0.0000–0.937]), and BFR/BS (baricitinib: 0.0037 [0.0018–0.1187], vehicle: 0.0015 [0.0000–0.00526]).

## 3. Discussion

Aging is associated with a chronic, often initially subclinical, proinflammatory state termed inflammaging. A hallmark of inflammaging is the sustained overproduction of proinflammatory cytokines—many of which are also bone-resorptive—thereby promoting osteoclast-driven bone loss and contributing to age-related disorders such as RA, and particularly osteoporosis [8,31,32,33]. In recent years, JAK inhibitors have emerged as a promising therapeutic approach for inflammatory diseases, such as RA, by targeting JAK-mediated cytokine signaling. Tighter control of inflammation through cytokine inhibition may not only halt, but also partially reverse, inflammatory bone damage.

This study aimed to investigate the bone-active effect of baricitinib, a selective JAK1 and JAK2 inhibitor, in a mouse model for age-related osteoporosis. We demonstrated that baricitinib suppressed the production of key proinflammatory cytokines, potentially resulting in a pronounced attenuation of inflammatory responses. In this context, we specifically examined IL-6, TNFα, IL-17, IL-21 and IFN-γ since these cytokines are not only central drivers of inflammation, but also have a well-documented impact on bone metabolism.

Proinflammatory cytokines such as TNFα, IL-6, and particularly IL-17 are elevated in the synovial fluid of patients with RA and promote bone destruction by stimulating osteoclast differentiation and/or activation [34,35]. These cytokines are also thought to play a key role in the pathogenesis of osteoporosis, as seen in experimental animal studies [34]. Moreover, IL-21 may further contribute to the inflammatory milieu in RA by activating T cells and promoting the secretion of proinflammatory cytokines, including TNFα and IFN-γ [36].

Evidence for a cytokine-driven component of osteoporosis comes from studies showing increased levels of TNFα, IL-1, IL-6, and IL-17 in the first ten years after menopause and in postmenopausal women with osteoporosis, relative to those without osteoporosis [37,38]. In line with this, D’Amelio et al. linked estrogen deficiency to enhanced osteoclastogenesis through increased production of TNFα and RANKL, while Zhao et al. found higher circulating levels of IL-17, IL-6, TNFα, and OPG in osteoporotic postmenopausal women than in controls [38,39].

The strength of our study was the direct assessment of immune-related cytokine profiles in splenic immune cell populations; this novel aspect is complementary to the study by Adam et al. [15] that evaluated the effect of baricitinib treatment in OVX and STA mice, with the main focus given to bone microarchitecture. Our results showed significantly decreased IL-6 levels in both CD4^+^ T cells and CD8^+^ T cells in baricitinib-treated SAMP8 mice compared to the vehicle-treated group. A decrease in IL-17, IFN-γ and IL-21 was observed only in CD4^+^ T cells under the treatment with baricitinib. Baricitinib and other JAKis are thought to mediate part of their therapeutic benefit in RA through attenuation of IL-6 signaling. Another study confirmed that baricitinib inhibits angiogenesis in RA synovial tissue and suppresses IL-6-induced inflammation [40]. Consistently, Yaekura et al. demonstrated in a collagen-induced arthritis (CIA) mouse model that baricitinib reduced IL-17A expression in splenic lymphocytes [41]. In line with our findings, studies in patients with COVID-19 reported that baricitinib reduced proinflammatory cytokines such as IL-1β, IL-6, TNFα, IFN-γ, and IL-17, while also affecting B-cell and T-cell frequencies [42,43]. The cited studies demonstrate the anti-inflammatory effects of baricitinib and are in line with our observation of reduced cytokine levels in SAMP8 mice. Importantly, Ding et al. reported that variation even within low circulating levels of inflammatory markers, particularly IL-6, predicted subsequent bone loss and increased resorption, suggesting that targeted anti-inflammatory strategies may help prevent osteoporosis [34]. To our knowledge, baricitinib has not yet been evaluated in a mouse model of age-related osteoporosis.

Looking at the microarchitectural bone effects, our results showed no significant differences in trabecular BV/TV of the femur, nor in the vertebra, when comparing SAMP8 mice treated with 10 mg/kg baricitinib and vehicle-treated mice. Adam et al. [15] reported that under steady-state conditions, young C57BL/6 mice treated with 50 mg/kg tofacitinib exhibited a significantly higher trabecular bone mass in the tibia compared to the vehicle-treated group. Moreover, the group tested the osteoanabolic effect of JAKis tofacitinib 50 mg/kg and 10 mg/kg baricitinib under noninflammatory (ovariectomized (OVX) mice) and inflammatory (serum-transfer arthritis (STA) mice) conditions. In the OVX setting, baricitinib raised trabecular bone mass in the tibia and vertebra. In the arthritic mice, µCT analysis of the tibia also showed a higher trabecular bone density as well than in control mice, mainly due to greater trabecular thickness. Furthermore, in OVX mice, treatment with both inhibitors led to a pronounced increase in trabecular thickness in the tibia and vertebra. Our study showed a non-significant trend towards a more favorable bone microarchitecture in the femur based on BV/TV, Tb.N and Tb.Sp parameters in the baricitinib group. Under steady-state conditions, young C57BL/6 mice either did not exhibit any significant changes in the vertebra or no alterations in the Tb.N in the tibia when treated with tofacitinib [15]. Cortical bone parameters were not significantly altered in the femur of baricitinib-treated SAMP8 mice compared to the vehicle-treated group, which was also observed by Adam et al. [15] in young C57BL/6 and OVX mice both in the tibia as well as the spine. Conversely, STA mice displayed a higher cortical thickness under tofacitinib and baricitinib treatment, whereas baricitinib also led to an increased cortical area fraction compared with vehicle-treated mice [15]. In a study by Schulz et al., a one-year treatment with baricitinib in patients with RA showed no significant changes in the BMD of the lumbar spine or femoral neck [28]. To explore a potential baricitinib-dependent effect on the BMD, a subgroup analysis was performed comparing treatment responders and non-responders. This represented a statistically significant deterioration of lumbar spine BMD in the non-responder group, while responders exhibited an improvement of the BMD in both spine and femur without reaching statistical significance [28]. Schulz et al. demonstrated that baricitinib did not just contribute to a stabilization of the BMD loss, but also led to significantly reduced disease activity in patients (mean age 62 years) with RA [28]. The effect of baricitinib on BMD described by Schulz et al. is in line with our findings, in that neither study demonstrated a significant improvement in bone microarchitecture under baricitinib treatment. The slightly higher BV/TV observed in the femur of baricitinib-treated SAMP8 mice suggests that baricitinib may exert at least a modest protective effect against bone loss in this model. However, vertebral data were less consistent: vehicle-treated mice showed a non-significant trend toward higher Tb.N, while baricitinib-treated animals displayed a slight increase in Tb.Sp. These findings indicate that further studies are required to conclusively define the impact of baricitinib on bone architecture in a mouse model for age-related osteoporosis. In line with Schulz et al. [28], the BARE BONE study [24] showed that 4 mg baricitinib over 52 weeks achieved a significant improvement in the bone quality and functional properties of patients with RA (mean age 54 years). Disease activity and synovial inflammation were also improved, and BMD in the radial bone was stabilized after 1 year of treatment [24]. Komagamine et al. demonstrated that JAK inhibition attenuated systemic bone loss; more precisely, the reduction in bone volume and trabecular thickness of the lumbar vertebrae in CIA mice. Joint erosion and periarticular osteopenia were likewise ameliorated by administration of upadacitinib [14]. In a preclinical study that characterized the efficacy of baricitinib, treatment improved disease activity across three arthritis models (rAIA, CIA and CAIA, the collagen Ab-induced arthritis), with radiographic evidence of reduced structural joint damage in rAIA and suppression of histological joint inflammation and destruction in CAIA [27]. Taken together, JAK effects on bone appear variable. Bone sites and the age of the animals seem to influence responsiveness. Of note, Adam et al. [15] and Komagamine et al. [14] used younger mice than in our study (age ranged from 10 to 16 weeks across models). Unexpectedly, JAK inhibition by baricitinib in our model of accelerated aging had no consistent effects on bone properties. Both in aging rodents and humans, bone regeneration is markedly impaired. Examples for these alterations include the senescence of bone marrow stromal cells: their impaired differentiation into bone-forming cells leads to decreased osteoblast numbers and reduced bone formation (for review see Zhang et al. [44] and Gelles et al. [6]). We therefore hypothesize that the aforementioned aging processes could have impeded the beneficial effects of the improved inflammatory milieu. The possibility exists that with a higher dose or a longer treatment duration a more pronounced effect of baricitinib would have been observed. Beyond treatment-related factors, bone remodeling processes might differ between young and aged animals due to potential age-related alterations in JAK/STAT signaling, variations in responsiveness of osteoblasts and osteoclasts, and baseline inflammatory status. Moreover, reduced osteoblast responsiveness might be linked to pronounced cellular senescence observed in SAMP8 mice. We would like to indicate that the non-profound effects observed in our study require confirmation in studies with larger sample sizes and/or modified study design to further evaluate the effect of baricitinib on age-related osteoporosis. The SAMP8 model corresponds to a senile state with slower remodeling rates and thus may exhibit a delayed or attenuated response to treatment. Nevertheless, we also cannot exclude the possibility that in our model, inflammatory mechanisms play only a minor role in the pathogenesis of bone loss.

Histomorphometric analysis of the vertebral body revealed a significantly increased BV/TV in baricitinib-treated SAMP8 mice compared to the vehicle-treated group. The inconsistency in BV/TV observed between the two techniques may reflect both the use of different vertebral bodies and the distinct analytical approaches employed. Specifically, µCT analysis determined BV/TV in the fourth vertebral body (which had been pre-defined as the primary variable) using a three-dimensional, automated assessment of the whole vertebra, while histomorphometric analysis was carried out in the fifth vertebral body on a single, manually evaluated two-dimensional microscopic section. These methodological and anatomical differences may have contributed to the observed divergence in BV/TV values; nevertheless, we cannot exclude a chance effect (type I error). Neither osteoblast nor osteoclast numbers were significantly altered in SAMP8 mice under JAK inhibition. Adam et al. [15] did not detect any change in numbers of osteoblasts and osteoclasts in tibial sections of young C57BL/6 mice and OVX mice either, whereas arthritic mice revealed increased osteoclast numbers. Komagamine et al. observed a reduction in osteoclast numbers across three types of bone destruction in CIA mice treated with a JAK inhibitor [14].

PINP and CTX are recommended by the International Osteoporosis Foundation (IOF) and the International Federation of Clinical Chemistry and Laboratory Medicine (IFCC) as reference bone turnover markers, reflecting bone formation and bone resorption, respectively, and enabling better comparability across studies [45,46]. In the present study, serum PINP did not show a significant difference between the treatment group and vehicle group, indicating that systemic bone formation, as captured by PINP, was not markedly shifted by treatment during the study period. While group differences were not significant, a subset of mice showed higher CTX values, suggesting increased bone resorption in some animals. Guo et al. reported that serum CTX-I and PINP changed significantly over time in OVX rats, indicating that estrogen deficiency accelerates both bone resorption and formation and thereby increases overall bone turnover [47]. In patients with RA, JAK inhibition with tofacitinib has been associated with decreased CTX after 6 months of treatment, whereas PINP was not affected by tofacitinib [48].

Overall, our data demonstrate a reduction in the levels of proinflammatory cytokines upon baricitinib treatment in a mouse model of accelerated aging. The effect of baricitinib on the bone properties of SAMP8 mice was not pronounced. The immune-skeletal crosstalk and/or the anabolic response of osteoblasts to the improved inflammatory milieu following JAK inhibition might be impaired due to age-related alterations.

## 4. Materials and Methods

### 4.1. Animals

In this study, 60 female three-month-old SAMP8 (*SAMP8*/*TaHsd*) were purchased from Envigo, Venray, Netherlands. To reflect the higher prevalence of osteoporosis in postmenopausal women, only female mice were used in this study. Mice were housed at the animal facility of the Institute of Pathophysiology and Allergy Research, Medical University of Vienna, Austria, where they were subjected to a 12 h light and dark cycle, with free access to water and food (LASQCdiet^®^ Rod 16, Auto; LASvendi GmbH, Soest, Germany). The following measures were taken to minimize distress caused to the mice: the mice were euthanized when their food intake was severely restricted, their mobility was significantly reduced, and they had lost more than 20% of their body weight. Weight monitoring was performed in weekly intervals. The study was approved by the committee of animal experimentation (iTVK) at the Medical University of Vienna (number 2022-0.276.661). A total of 60 SAMP8 mice were initially enrolled. Prior to initiation of baricitinib treatment, three mice from different cages died of unknown causes. At eight months of age, mice were randomly divided into a treatment and control group. A total of 27 mice were treated twice-daily with 10 mg/kg body weight baricitinib (6.73 mM; MedChemExpress; 1187594-09-7, Monmouth Junction, NJ, USA) dissolved in 0.5% methylcellulose and 0.025% Tween 20 solution, and 30 mice received a vehicle (0.5% methylcellulose and 0.025% Tween 20 solution). Administration was completed via oral gavage for six weeks. The dosage and mode of administration of baricitinib was based on a study by Adam et al. [15]. The study was not conducted in a blinded manner. During the treatment period, two additional mice were lost due to acute health issues that were considered unrelated to baricitinib administration. A total of 55 animals completed the study and were included in the final analysis, except stated otherwise.

### 4.2. Flow Cytometry

For the analysis of cytokines, spleens from SAMP8 mice were dissected and minced to collect cells in medium (MEM Alpha Medium, Gibco, Waltham, MA, USA, 22561-021). Tubes containing Ficoll^®^ Paque PLUS (Merck, Darmstadt, Germany, GE17-1440-02) were prepared and cells in suspension were carefully pipetted on the top of the Ficoll-Paque. Leukocytes were obtained by density gradient centrifugation, aspirated, and resuspended in fresh medium (MEM Alpha Medium, Gibco, Waltham, MA, USA, 22561-021). Cells were counted and seeded in a density of 2 × 10^6^/mL cells for stimulation in 6-well plates. For the determination of intracellular cytokine production, cells were incubated for 4 h at 37 °C in culture medium (see above) with the following additives based on the protocol of Pietschmann et al. [49]: phorbol 12-myristate 13-acetate (50 ng/mL; Merck, P-8139), ionomycin (1.05 µg/mL; Sigma-Aldrich, St. Louis, MO, USA, I-0634), brefeldin A (5 µg/mL; BioLegend, San Jose, CA, USA, 420601), and monensin (2.0 mM; BioLegend, San Jose, CA, USA, 420701). Subsequently, cells were harvested, washed with 1× phosphate-buffered saline (PBS), centrifuged, resuspended in PBS, and incubated with 4% formaldehyde (Roti^®^ Histofix, Carl Roth, Karlsruhe, Germany, P087.3) for 20 min at room temperature (RT). After incubation, cells were centrifuged, washed and resuspended in PBS for staining of intracellular cytokines. Cells were centrifuged and permeabilized by washing twice with Perm/Wash (Intracellular Staining Permeabilization Wash Buffer 10×; BioLegend, 421002). Meanwhile, different combinations of antibodies were prepared in Perm/Wash. Cells were resuspended in Perm/Wash containing the corresponding antibodies and incubated for 25 min at RT. After incubation, cells were resuspended in PBS and analyzed using FACSCanto^TM^ II (BD FACSCanto^TM^ II Clinical Flow Cytometry System, BD Biosciences, Franklin Lakes, NJ, USA). The antibodies used for flow cytometry analysis are listed in Table 1. The corresponding fluorophore for markers of interest was chosen based on the marker combination in the particular staining setting. For data analysis, FlowJo^TM^ version 10 software (BD Biosciences, NJ, USA) was used.

The following gating strategy was applied: living cells were first gated on the basis of SSC-A and FSC-A; in the next step, the cells were gated based on CD4, CD8, and CD19 expression for CD4^+^, CD8^+^, and CD19^+^ populations, respectively. The inclusion criteria for the follow-up analysis of splenic cells were >5000 living cells and >200 cells for the individual populations, such as CD4^+^, CD8^+^, and CD19^+^ cells; the inclusion criteria for bone marrow cells were >2500 living cells. The analysis of the intracellular cytokine levels included the evaluation of IL-6, TNFα, IL-17, IL-21, and IFN-γ. The frequency of corresponding cell populations (in %) and of cytokines (in %), as well as the MFI were used as variables for follow-up statistical analysis. Initial experimental set up also included the corresponding analysis of the bone marrow of the femur. Due to insufficient cell number, sophisticated analysis could not be performed.

### 4.3. Enzyme-Linked Immunosorbent Assay

For determination of bone turnover markers, blood was obtained by cardiac puncture immediately after euthanasia, and serum was collected by centrifugation. A marker for bone formation—PINP—and a marker for bone resorption—CTX—was selected to investigate the effect of baricitinib on bone turnover [50]. Enzyme-linked immunosorbent assays (ELISAs; Immunodiagnostic Systems IDS^®^, Boldon, UK) for PINP (Rat/Mouse PINP EIA) and CTX (Rat-Laps^®^ EIA) were performed following the respective assay protocol by IDS^®^. Serum concentrations of PINP, diluted 1:10 in PBS, and CTX, undiluted, were measured.

### 4.4. Micro-Computed Tomography

For investigation of the cortical and the trabecular part of the bones, the femur and fourth vertebral body were analyzed by micro-computed tomography (µCT) using a microCT-35 device (Scanco Medical, Brütisellen, Switzerland). Samples were fixed in 4% formaldehyde (Roti^®^ Histofix, Carl Roth, Karlsruhe, Germany, P087.3) and stored in 70% ethanol. For the measurement, samples were rolled up in foam and placed in a specific holder during the scans. To avoid drying of the samples, the foam was soaked in 70% ethanol. The X-ray tube was set to 70 kV with an intensity of 114 µA and an integration time of 800 ms. These settings led to a resolution of 10 µm/pixel. Trabecular microstructure was evaluated in the distal femur and the lumbar spine (L4). In the femur, trabecular structure was analyzed proximally from the epiphysis across 10% of the total length of the femur, starting at 75% of its overall length. The cortical morphometry was determined over a length of 5% proximal and 5% distal from the midshaft of the whole femur length. Both the fourth lumbar vertebra and the femur were manually delineated. For the separation of bone and nonbone tissue, thresholds of 260 and 220 hydroxyapatite/cm^3^ (HA/cm^3^) were applied. For the evaluation of the scans, the Xming^TM^ program was used. Cortical and trabecular microstructural parameters were evaluated according to the guidelines of the American Society for Bone and Mineral Research (ASBMR) [51]. Cortical parameters such as Ct.Ar/Tt.Ar, Ct.Ar, Tt.Ar, and Ct.Th were assessed. The evaluation of trabecular parameters included BV/TV, Tb.Th, Tb.N and Tb.Sp. The selected microstructural parameters were automatically calculated by the aforementioned program. Representative images are shown in Supplementary Figure S2 (Aa, Ab, Ba, Bb).

### 4.5. Bone Histomorphometry

In this study, dynamic and static bone histomorphometry were performed. For dynamic bone histomorphometry, mice received intraperitoneal injections of 20 mg/kg body weight calcein (C0875, Sigma) 10 and 3 days before euthanasia based on a protocol by Rauner et al. [50]. The third lumbar vertebra was fixed in 4% formaldehyde (ROTI^®^ Histofix, Carl Roth^®^, P087.3) and subsequently dehydrated using an ascending ethanol series. The undecalcified vertebral bodies were embedded in methyl methacrylate (K-Mount Powder Component, DiaTec, Oslo, Norway) and cut in 11 µm thick sections with the Microtome Microm HM355S (Thermo Scientific, Waltham, MA, USA). For the assessment of fluorescent calcein labels, MS/BS, MAR and BFR/BS was determined. A representative image is shown in Supplementary Figure S2C. For the evaluation of static bone histomorphometry, the fifth lumbar vertebra was fixed, decalcified in Tris EDTA and dehydrated as described above, followed by embedding in paraffin (Paraffin 54-56, Carl Roth^®^, 6642.5). Tartrate-resistant acid phosphatase (TRAP) (Sigma-Aldrich, St. Louis, MO, USA) and toluidine blue (Fluka Analytical, Sigma-Aldrich, St. Louis, MO, USA) staining was performed on 5 µm thick paraffin sections for the identification of osteoblasts and osteoclasts. A representative image is shown in Supplementary Figure S2C. N.Ob/B.Pm, N.Oc/B.Pm, N.Ot/B.Ar and ES/BS were determined, as well as trabecular parameters including BV/TV, Tb.N, Tb.Th and Tb.S. Dynamic and static histomorphometric analyses were performed as blinded experiments using the Osteomeasure^TM^ system (OsteoMetrics, Decatur, GA, USA) according to the guidelines of the ASBMR histomorphometry nomenclature committee [52].

### 4.6. Statistical Analysis

Sample size calculations were performed by Prof. Dr. Florian Frommlet (Institute of Medical Statistics, Center for Medical Statistics, Informatics and Intelligent Systems, Medical University of Vienna) and were based on previously published μCT data by Adam et al. [15]. Assuming a 20% attrition rate over the six-week treatment period, we expected 48 mice to reach the end of the experiment. This corresponded to 24 mice per group, which ensured 90% power to detect a 5.0% difference in vertebral BV/TV between groups (SD 5.0%), based on a two-sided Satterthwaite *t*-test at α = 0.05. All results were tested for normality using the Shapiro–Wilk test. For statistical evaluation, normally distributed data were analyzed using unpaired *t*-tests, whereas for non-normally distributed data, Mann–Whitney tests were used. Results with *p* < 0.05 were considered statistically significant. GraphPad Prism version 10 (Dotmatics, Boston, MA, USA) was used for analysis.

## 5. Conclusions

In conclusion, our data demonstrate that baricitinib in a mouse model of accelerated aging reduces the levels of proinflammatory cytokines, including IL-6, IL-17, IL-21, and IFN-y as well as of the TNFα/IL-6, IFN-γ/IL-17, and IL-21/IL-6 cytokine combinations. This supports its potential use as a therapeutic regimen for age-related inflammatory diseases, such as late-onset rheumatoid arthritis. Despite this, no consistent effects of baricitinib on bone properties were observed. These findings suggest an altered responsiveness of bone to JAK inhibition with age and indicate that the potential of baricitinib as a standalone anabolic therapy for senile osteoporosis may be limited.

## Figures and Tables

**Table 1:** List of antibodies used for the FACS analysis.

Antibody	Conjugation	Isotype	Clone	Company
CD4	APC	Rat (DA) IgG2a, κ	RM4-5	BD Pharmingen, San Diego, CA, USA
CD8	Alexa Fluor^®^ 700	Rat (LOU) IgG2a, κ	53–6.7	BD Pharmingen, San Diego, CA, USA
CD8a	PE-Cy^TM^7	Rat (LOU) IgG2a, κ	53–6.7	BD Pharmingen, San Diego, CA, USA
CD19	PE	Rat (LEW) IgG2a, κ	1D3	BD Pharmingen, San Diego, CA, USA
TNFα	PerCP-Cy^TM^5.5	Rat (LEW) IgG2a, κ	MP6-XT22	BD Pharmingen, San Diego, CA, USA
IL-6	Alexa Fluor^®^ 488	Rat IgG1	MP5-20F3	BD Pharmingen, San Diego, CA, USA
IL-17	PerCP-Cy^TM^5.5	Rat (LEW) IgG2a, κ	TC11-18H10	BD Pharmingen, San Diego, CA, USA
IL-21	PE	Rat/IgG2a, κ	FFA21	Invitrogen, Waltham, MA, USA
IFN-γ	PE-Cy^TM^7	Rat IgG1, κ	XMG1.2	BD Pharmingen, San Diego, CA, USA

## Data Availability

The data that support the findings of this study are included in the article/Supplementary Material; further inquiries can be directed to the corresponding authors.

## Supplementary Materials

The following supporting information can be downloaded at: https://www.mdpi.com/article/10.3390/ijms27115047/s1.

## Abbreviations

The following abbreviations are used in this manuscript:

JAK	Janus kinase
SAMP8	Senescence-accelerated mice-prone 8
µCT	Micro-computed tomography
IL	Interleukin
TNF	Tumor necrosis factor
IFN	Interferon
STAT	Signaling transducer of activators of transcription
TYK2	Tyrosin kinase 2
JAKi	Janus kinase inhibitor
RANKL	RANKL receptor for activation of nuclear factor kappa B (NF-*κ*B) ligand
BMD	Bone mineral density
MFI	Mean fluorescence intensity
PINP	p-terminal propeptide of type I procollagen
CTX	c-terminal telopeptide of type I collagen
Ct.Th	Average cortical thickness
Ct.Ar/Tt.Ar	Cortical bone area fraction
Ct.Ar	Cortical bone area
Tt.Ar	Total cross-sectional area inside the periosteal envelope
BV/TV	Bone volume fraction
Tb.N	Trabecular number
Tb.Th	Trabecular thickness
Tb.Sp	Trabecular separation
MAR	Mineral apposition rate
MS/BS	Mineralized surface per bone surface
BFR/BS	Bone formation rate per bone surface
N.Ob/B.Pm	Number of osteoblasts per bone perimeter
N.Oc/B.Pm	Number of osteoclasts per bone perimeter
N.Ot/B.Ar	Number of osteocytes per bone area
ES/BS	Eroded surface/bone surface
CIA	Collagen induced arthritis
OVX	Ovariectomy
STA	Serum-transfer arthritis
IOF	International Osteoporosis Foundation
IFCC	International Federation of Clinical Chemistry and Laboratory Medicine
PBS	Phosphate-buffered saline
RT	Room temperature
EDTA	Ethylenediaminetetraacetic acid
TRAP	Tartrate-resistant acid phosphatase
ELISA	Enzyme-linked immunosorbent assays
Wnt	Wingless-related integration site
