# Supplementary material for: Effect of the JAK Inhibitor Baricitinib on Cytokine Production and Bone Properties in a Mouse Model of Accelerated Aging
Source: Int J Mol Sci. 2026 Jun 3;27(11):5047. doi: 10.3390/ijms27115047 (PMC13256813; doi:10.3390/ijms27115047)
Supplement: Supplementary file 1 [file ijms-27-05047-s001.zip › ijms-4295793-supplementary.pdf]

Supplementary

Effect of the JAK Inhibitor Baricitinib on Cytokine Production and Bone Properties in a Mouse Model of Accelerated Aging

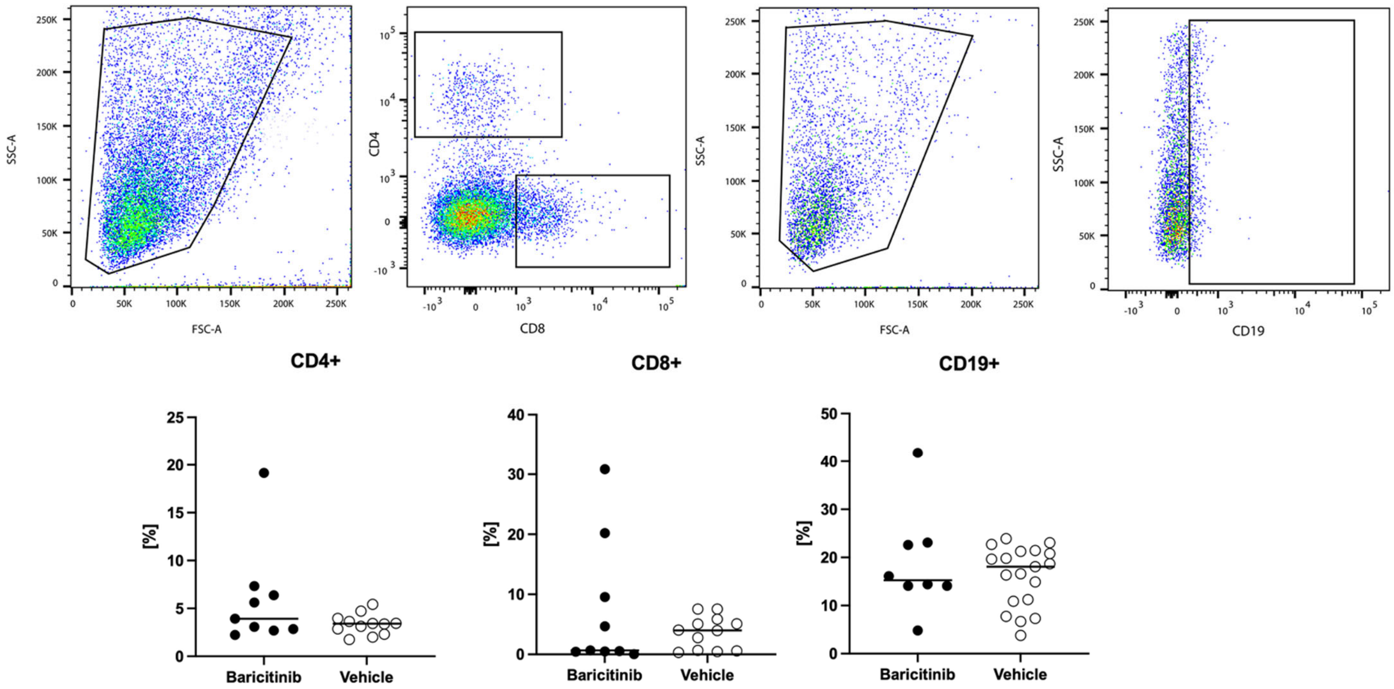

Supplementary Figure S1. Gating strategy and frequencies of CD4⁺, CD8⁺, and CD19⁺ cells in the bone marrow. Representative dot plots showing the gating strategy are presented together with the frequencies of CD4⁺, CD8⁺, and CD19⁺ cells in the baricitinib-treated and vehicle-treated groups. The gating strategy and inclusion criteria are detailed in Section 4. Pseudocolor plots are used to visualize the density of cell population(s). Because the data were not normally distributed, the Mann–Whitney test was used. Horizontal lines represent the median.

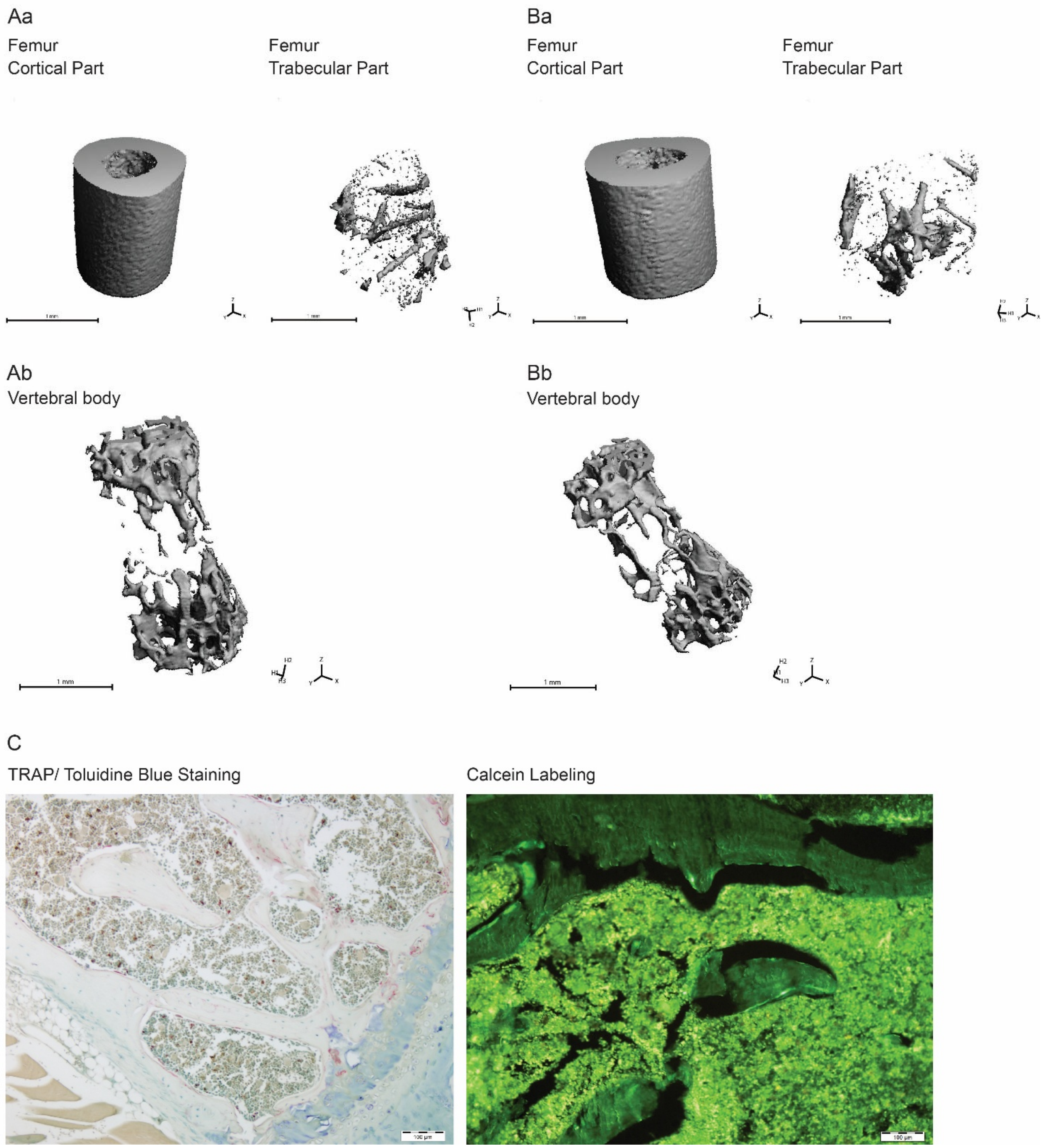

Supplementary Figure S2. Representative images of µCT analysis and of histological staining of bone tissue. Shown are representative images of the µCT analysis of the cortical and trabecular region of the femur ((**Aa**), baricitinib-treated SAMP8 mouse; (**Ba**), vehicle-treated SAMP8 mouse) and the trabecular part of the fourth vertebral body ((**Ab**), baricitinib-treated SAMP8 mouse; (**Ba**), vehicle-treated SAMP8 mouse). Representative images of TRAP/toluidine blue staining of the fifth lumbar vertebra and calcein labeling of the third vertebral body are shown (**C**). Scale bars are indicated in the respective images.
